# Postoperative Aqueous Flare in Diabetic Eyes and Fluidics-Related Surgical Parameters After Cataract Surgery Using the Eight-Chop Technique

**DOI:** 10.3390/jcm15176547

**Published:** 2026-08-25

**Authors:** Tsuyoshi Sato

**Affiliations:** Department of Ophthalmology, Sato Eye Clinic, Nemoto 3-3, Matsudo-Shi 271-0077, Chiba-Ken, Japan; perfect-eightchop@sato-ganka.com

**Keywords:** aqueous flare, cataract, diabetes mellitus, Eight-Chop Technique, phacoemulsification

## Abstract

**Objectives**: This study aimed to quantify aqueous flare in diabetic eyes after low-stress phacoemulsification and identify factors associated with persistent inflammation. **Methods**: Eyes undergoing active-fluidics phacoemulsification with the Eight-Chop Technique were assigned to diabetes mellitus (DM) or nondiabetic control groups. Aqueous flare was measured by laser flare photometry preoperatively and at postoperative day 1, day 7, week 7, and week 19. Intraoperative variables (cumulative dissipated energy [CDE], irrigation fluid volume, and operative time) and diabetic retinopathy status were recorded. Mixed-effects models evaluated longitudinal flare changes; multivariable regression identified factors for day 7 flare. **Results**: A total of 172 eyes (86 DM; 86 controls) from 106 patients were analyzed. Postoperative flare increased in both groups, peaked on day 7, and then declined; diabetic eyes had significantly higher flare throughout. Day 7 flare was higher in diabetic eyes (26.4 vs. 16.3 ph/ms), approximately 39% higher in a patient-clustered analysis (95% CI: 14–70%; *p* = 0.001). After adjusting for preoperative flare, irrigation fluid volume, CDE, and operative time, diabetic eyes had approximately 25% higher day 7 flare (95% CI: 3–53%; *p* = 0.026), persisting across time points (interaction *p* = 0.087), with similar results in a complete-case sensitivity analysis. Within this model, preoperative flare and irrigation fluid volume were significantly associated with higher day 7 flare, whereas cumulative dissipated energy and operative time were not. **Conclusions**: Under low-stress phacoemulsification, diabetic eyes exhibited persistently higher postoperative aqueous flare. Day 7 flare may indicate persistent barrier dysfunction, and its association with irrigation fluid volume suggests fluidics-related factors in inflammation.

## 1. Introduction

Cataract surgery is one of the most frequently performed ophthalmic procedures worldwide, and advances in phacoemulsification technology have substantially improved surgical safety and visual outcomes [1]. Nevertheless, postoperative inflammation remains an important concern, particularly in eyes with diabetes mellitus (DM), in which blood–aqueous barrier (BAB) dysfunction may predispose to prolonged intraocular inflammation and postoperative macular complications [2,3,4,5]. Laser flare photometry (LFP) provides an objective quantitative method for evaluating BAB disruption and has become an established tool for assessing postoperative anterior chamber inflammation [3,4,6,7,8,9,10].

Liu et al. reported that diabetic eyes exhibited significantly higher postoperative aqueous flare values than nondiabetic eyes after phacoemulsification, particularly in the presence of diabetic retinopathy (DR) [2]. Persistent flare elevation at postoperative day 7 was also observed, suggesting that this time point may be clinically important for evaluating postoperative inflammatory responses in diabetic eyes [2,4,5]. Since those earlier reports, cataract surgery has evolved substantially, with improvements in phacoemulsification efficiency, fluidics control, active-fluidics systems, and small-incision techniques leading to less invasive surgery [11,12,13]. However, it remains unclear to what extent postoperative inflammatory responses persist in diabetic eyes under current low-stress phacoemulsification conditions and how contemporary fluidics-related factors contribute beyond ultrasound energy alone.

The Eight-Chop Technique is a “segmentation-first” nuclear fragmentation strategy that achieves complete full-thickness nuclear division before phacoemulsification [12,14]. This wedge-based fracture approach aims to reduce ultrasound energy usage, improve fragment controllability, optimize fluidics stability, and minimize mechanical stress on intraocular tissues, including the posterior capsule and zonular apparatus; reduced dependence on a second instrument may further contribute to stable anterior chamber conditions [12,14].

Postoperative inflammatory responses after cataract surgery using the Eight-Chop Technique in diabetic eyes have not been fully characterized using LFP. In addition, intraoperative factors associated with postoperative inflammation under modern low-stress phacoemulsification—particularly fluidics-related factors such as irrigation fluid volume compared with conventional measures of surgical energy—remain incompletely understood. In this study, low-stress phacoemulsification was defined as surgery performed with modern active-fluidics systems and a segmentation-first Eight-Chop fragmentation strategy that aims to minimize ultrasound energy and fluidics-related stress. The purpose of the present study was to evaluate postoperative aqueous flare responses in diabetic eyes after cataract surgery using the Eight-Chop Technique and to identify factors associated with postoperative inflammation, with a specific focus on irrigation fluid volume and cumulative dissipated energy (CDE).

The primary hypothesis of this study was that diabetic eyes would exhibit a significantly higher aqueous flare at postoperative day 7 than nondiabetic eyes, reflecting persistent BAB dysfunction. Accordingly, the prespecified primary outcome measure was the between-group difference in aqueous flare at day 7 after surgery. Flare measurements at other postoperative time points (day 1, week 7, and week 19) were defined as secondary endpoints.

## 2. Materials and Methods

### 2.1. Ethical Considerations

This prospective observational study was approved by the institutional ethics committee of Sato Eye Clinic and conducted in accordance with the tenets of the Declaration of Helsinki (approval number: 2025010602). The study protocol was approved on 6 January 2025, and clinical data were collected prospectively from consecutive patients undergoing cataract surgery between 20 January 2025 and 2 December 2025, with postoperative follow-up data collected through 30 June 2026. The purpose of this study, including the use of clinical data for research, was explained to all patients before surgery. This study was conducted as a prospective observational cohort study; as it did not involve the assignment of an intervention, it was not registered in a clinical trial registry (e.g., UMIN-CTR, ClinicalTrials.gov). Written informed consent was obtained individually from each participant prior to enrollment.

### 2.2. Study Population

This study included consecutive patients who underwent phacoemulsification using the Eight-Chop Technique at Sato Eye Clinic (Matsudo City, Chiba Prefecture, Japan). Eyes were divided into a DM group and a nondiabetic control group according to the presence or absence of DM. Control eyes were selected as follows: 100 nondiabetic eyes were initially enrolled and followed postoperatively; 10 eyes were subsequently excluded because the patient did not attend one or more scheduled postoperative visits, resulting in missing data, including aqueous flare, at those time points. From the remaining 90 eyes with complete flare data at every time point, 86 eyes were selected to match the diabetic group on age and Emery–Little grade [15]; the remaining 4 eyes were not selected. These 4 eyes were identified as contributing most to the between-group imbalance in age relative to the diabetic group and were excluded to improve this approximation, with particular attention to avoiding the retention of eyes with harder nuclei (Emery–Little grades 3–4) that could disrupt the age approximation; selection was not based on individual pairwise (nearest-neighbor) matching to a specific diabetic eye. This yielded a final control group of 86 eyes with complete flare data at every postoperative time point. DR was classified based on fundus examination into no DR, simple DR, preproliferative DR, and proliferative DR. For multivariable analyses, we defined a binary variable “any DR,” which was coded as 1 for eyes with simple or preproliferative DR and 0 for eyes without DR. In this cohort, no eyes had simple DR, and 8 eyes had preproliferative DR; thus, only preproliferative DR contributed to the “any DR = 1” category. DR was assessed by a retina and vitreous specialist using dilated fundus examination and optical coherence tomography. Fundus photographs were reviewed when necessary to support clinical grading, but DR staging was based primarily on clinical examination and optical coherence tomography findings. Grading was performed by a single specialist without masking to diabetic status, within approximately 1 month before cataract surgery at a routine outpatient visit. Eyes with a history of retinal laser photocoagulation or intravitreal anti-vascular endothelial growth factor injections were not explicitly excluded; however, none of the eyes included in this study had a history of intravitreal anti-vascular endothelial growth factor therapy. Exclusion criteria were corneal disease or opacity; uveitis or glaucoma; retinal disease other than DR; congenital ocular anomalies such as microcornea, nanophthalmos, or anterior segment dysgenesis; previous ocular trauma or intraocular surgery; Emery–Little Grade 4 or higher nuclei; cases requiring iris retractors or capsular tension devices; and intraoperative complications. Among diabetic eyes, in most cases, the patient did not attend the scheduled postoperative visit at one or more time points, resulting in missing data for all assessments performed at that visit, including aqueous flare; in a small number of cases, only some assessments, including in one instance aqueous flare alone, were not obtained despite the visit being attended. These eyes were not excluded and were retained in the primary analysis using all available measurements at the remaining time points (11 of 86 diabetic eyes had missing flare data at week 7, and a cumulative 20 of 86 at week 19). As noted above, completeness of aqueous flare data at every time point was an additional inclusion requirement specific to the control group. Both eyes were included when eligible. Because bilateral eyes from the same patient are not statistically independent, intra-patient correlation was accounted for using linear mixed-effects models in the statistical analysis.

Because this study was designed as a prospective observational investigation with consecutive enrollment of eligible eyes, no formal a priori sample size calculation was performed at study initiation. To evaluate whether the final sample size was adequate for the primary endpoint, we subsequently conducted a post hoc power assessment using the between-group difference in aqueous flare at postoperative day 7 as the primary outcome. Assuming an alpha level of 0.05 (two-sided), a standard deviation of approximately 9.0 ph/ms in nondiabetic eyes and 25.9 ph/ms in diabetic eyes based on the observed data, and an expected difference of about 10 ph/ms between the mean day-7 flare values of the two groups (16.3 ± 9.0 ph/ms in controls vs. 26.4 ± 25.9 ph/ms in diabetic eyes; *n* = 86 eyes per group), the achieved sample size of 86 diabetic and 86 nondiabetic eyes provided a statistical power of approximately 80–90% to detect this difference. In contrast, subgroup analyses involving eyes with any DR (*n* = 8) were underpowered and are, therefore, interpreted as exploratory and hypothesis-generating.

**Figure 1:**
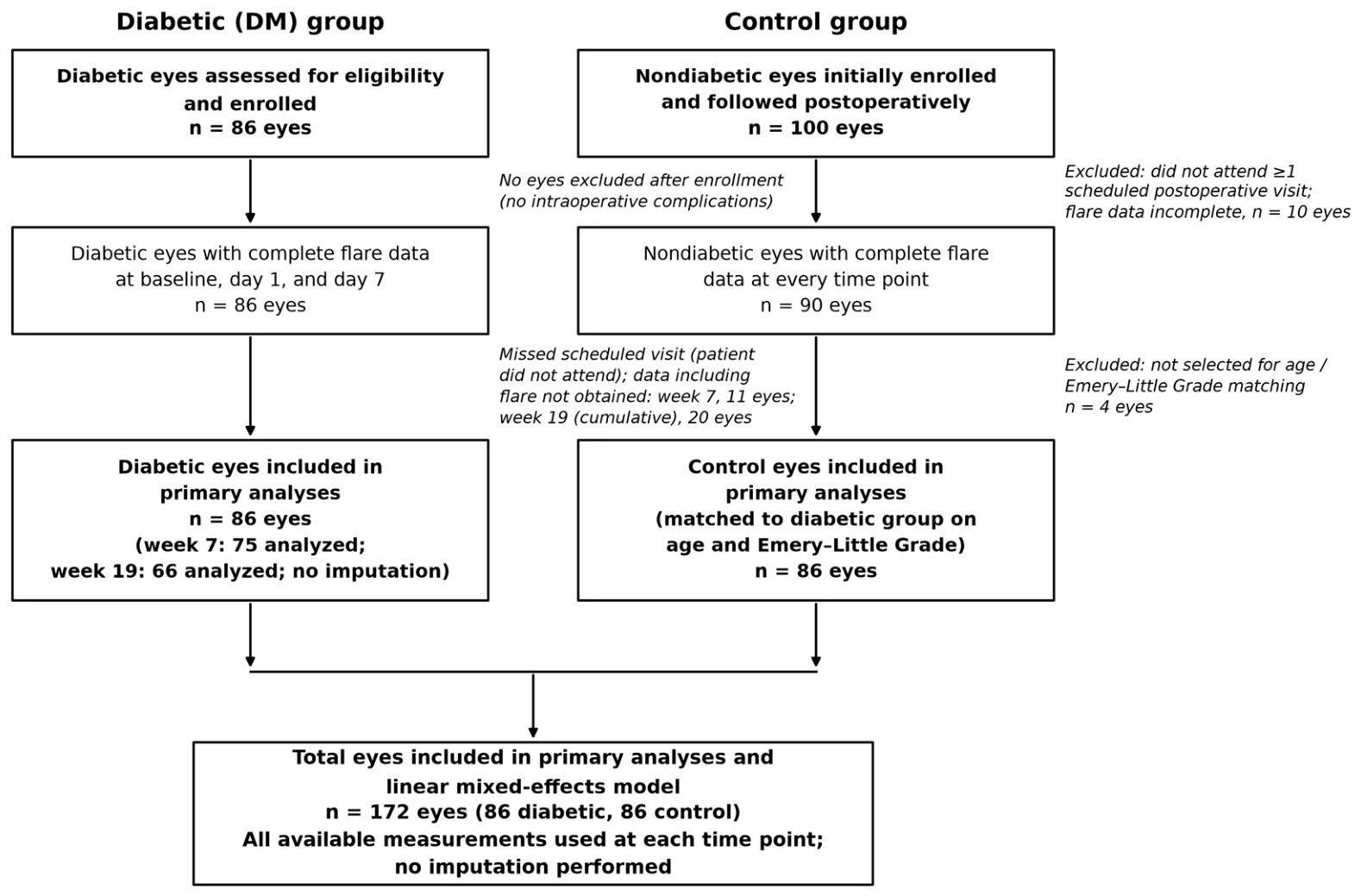
Flow diagram of eye selection, enrollment, and analysis for the diabetic and control groups. Diabetic eyes: A total of 86 eyes were enrolled and underwent cataract surgery with the Eight-Chop Technique; no eyes were excluded after enrollment. Control eyes: A total of 100 nondiabetic eyes were initially enrolled and followed postoperatively; 10 eyes were excluded because the patient did not attend one or more scheduled postoperative visits, resulting in missing aqueous flare data at those time points, and from the remaining 90 eyes with complete flare data at every time point, 86 eyes were selected to match the diabetic group on age and nuclear hardness (Emery–Little grade). All 86 diabetic eyes and all 86 control eyes were included in the primary analyses. Among diabetic eyes, some patients did not attend the scheduled visit at week 7 (11 eyes) or week 19 (cumulative, 20 eyes), resulting in missing data, including aqueous flare, at that time point; these patients continued to attend subsequent visits and were not excluded. No imputation was performed, and the linear mixed-effects model used all available measurements at each time point.

To address potential confounding by sex and the correlation between fellow eyes, we additionally fitted a linear mixed-effects model for postoperative day-7 flare including diabetic status, sex, and preoperative flare as fixed effects, with a random intercept for patient ID. This model accounted for the correlation between bilateral eyes within the same patient via the patient-level random intercept. The study flow, including eye selection, enrollment, and analysis, is shown in Figure 1.

### 2.3. Preoperative Assessment

All patients underwent comprehensive ophthalmic examination before surgery. Best-corrected visual acuity (BCVA) was measured using a decimal chart and converted to the logarithm of the minimum angle of resolution (logMAR). Intraocular pressure (IOP) was measured preoperatively. Corneal endothelial parameters, including corneal endothelial cell density (CECD), central corneal thickness (CCT), coefficient of variation (CV), and percentage of hexagonal cells (PHC), were obtained using a non-contact specular microscope (EM-3000; Topcon, Tokyo, Japan). Anterior chamber depth and axial length were measured using a sweep-source optical biometer (OA-2000; Tomey, Nagoya, Japan). Preoperative aqueous flare was measured using a laser flare photometer.

### 2.4. Aqueous Flare Measurement

Aqueous flare was measured using a laser flare photometer (FM-600; Kowa, Tokyo, Japan) under controlled lighting conditions by a total of four trained examiners following a standardized measurement protocol. Five consecutive measurements were obtained at each examination, and the mean value was used for analysis. Flare intensity was expressed as photon counts per millisecond (ph/ms), quantitatively reflecting protein concentration in the anterior chamber and the degree of BAB disruption.

### 2.5. Surgical Technique

All surgeries were performed by a single experienced cataract surgeon (T.S.) using the Eight-Chop Technique. A 3.0 mm clear corneal incision was created, followed by continuous curvilinear capsulorhexis of approximately 6.0–6.2 mm and hydrodissection. The lens nucleus was mechanically divided into eight segments using the Eight-Chopper II (SP-8402; ASICO, Parsippany, NJ, USA) before phacoemulsification, and each fragment was subsequently emulsified and aspirated using the Centurion Vision System (Alcon, Fort Worth, TX, USA). The system was operated with a target intraocular pressure of 55 mmHg, a maximum vacuum of 500 mmHg, and an aspiration flow rate of 32 mL/min. The active-fluidics pressure target was kept constant at 55 mmHg throughout phacoemulsification and irrigation/aspiration. Ultrasound was delivered in pulse plus linear mode with torsional ultrasound set to 0%, and a Flare ABS phaco tip with a 1.1 mm MicroSmooth sleeved configuration was used in all cases. The residual cortex was removed by irrigation/aspiration, and a foldable hydrophobic acrylic intraocular lens (AcrySof MN60AC; Alcon, Fort Worth, TX, USA) was implanted in the capsular bag. At the completion of surgery, viscoelastic material was thoroughly removed, and the anterior chamber was replenished with balanced salt solution containing moxifloxacin (0.5 mg/mL). Intraoperative parameters automatically recorded by the phacoemulsification system included phaco time (s), aspiration time (s), CDE, and irrigation fluid volume (mL). Operative time (min) was manually measured from corneal incision creation to completion of intraocular viscoelastic removal.

### 2.6. Postoperative Examinations

Postoperative examinations were performed at postoperative day 1, day 7, week 7, and week 19. At each visit, BCVA, IOP, aqueous flare, CECD, CCT, CV, and PHC were measured using the same instruments and protocols as those used preoperatively. Aqueous flare values were measured five consecutive times, and the mean value was used for analysis. CECD loss (%) was calculated using the following formula:

$$CECD\;loss\;(\%)=\frac{Preoperative\;CECD-Postoperative\;CECD}{Preoperative\;CECD}\times 100$$

### 2.7. Postoperative Medication Regimen

All patients received the same standardized postoperative anti-inflammatory and antibiotic regimen. Immediately after surgery, topical betamethasone 0.1% and moxifloxacin 0.5% were prescribed four times daily for 1 week. Thereafter, betamethasone was switched to fluorometholone 0.1%, and fluorometholone 0.1% plus moxifloxacin 0.5% were administered three times daily for an additional 4 weeks. No routine use of topical non-steroidal anti-inflammatory drugs was employed, and the postoperative regimen was not modified during the observation period.

### 2.8. Statistical Analysis

Statistical analyses were performed using R (version 4.3.2; R Foundation for Statistical Computing, Vienna, Austria). The normality of continuous variables was assessed using histograms, Q–Q plots, and the Shapiro–Wilk test when appropriate. Approximately normally distributed variables were analyzed using parametric methods, and non-normally distributed variables using nonparametric methods. Between-group comparisons were performed using Welch’s *t*-test for continuous variables and the chi-square test for categorical variables. Of the 106 patients included, 66 contributed both eyes to the analysis (33 in each group). Longitudinal changes in aqueous flare were evaluated using linear mixed-effects models with patient ID as a random intercept to account for repeated measurements and intra-patient correlation in bilateral cases. Some eyes had missing aqueous flare measurements at specific postoperative time points, predominantly due to missed scheduled visits unrelated to postoperative complications or adverse events (Section 3.3). No imputation (e.g., multiple imputation) was performed; linear mixed-effects models were fitted using only the available measurements under the assumption of missing at random. The primary analysis of factors associated with postoperative day 7 aqueous flare was a single prespecified linear mixed-effects model, with patient ID included as a random intercept to account for correlation between bilateral eyes (described in detail in Section 3.5). Covariates were selected a priori based on their hypothesized roles: DM group (primary exposure of interest), preoperative flare (baseline BAB status), irrigation fluid volume and CDE (intraoperative fluidics-related and ultrasound-related surgical stress, respectively), and operative time (overall surgical duration and complexity). This model constitutes the primary inferential analysis for postoperative day 7 flare in this study. Because aqueous flare values showed a markedly right-skewed distribution, flare measurements were log-transformed (natural logarithm) before this and all other regression analyses involving flare as an outcome; log-transformed postoperative day 7 flare was used as the outcome variable in the primary mixed-effects model. To assess potential multicollinearity among predictors in this model, variance inflation factors (VIFs) were calculated for each explanatory variable. Group comparisons and descriptive statistics (Tables 1–4) were based on raw flare values expressed in ph/ms. The between-group comparisons in Tables 1–4 are descriptive baseline, surgical, and safety-parameter comparisons and were not adjusted for clustering between bilateral eyes; correlation between fellow eyes was accounted for exclusively in the mixed-effects models for aqueous flare described above and in Section 3.5 and Section 3.6. Model assumptions for the regression and mixed-effects analyses were assessed using residual plots and Q–Q plots; these diagnostics did not reveal major deviations from normality or homoscedasticity. Variance components and the intraclass correlation coefficient (ICC) from the mixed-effects model are reported in the Results. Regression coefficients, standard errors, and *p*-values were obtained using Satterthwaite’s approximation. All tests were two-sided, and *p* < 0.05 was considered statistically significant. Data are presented as means ± standard deviation unless otherwise specified. Representative model formulas and R code snippets for the regression and mixed-effects models are provided in Supplementary File S2.

To evaluate whether the between-group difference in aqueous flare changed over time, a linear mixed-effects model was fitted to natural-log-transformed flare values at all five time points (preoperative, day 1, day 7, week 7, and week 19), with fixed effects for group, time, and their interaction, and a random intercept for patient ID to account for correlation between bilateral cases. A three-level structure additionally nesting eye within patient was also tested but did not improve model fit (ΔAkaike Information Criterion = 2.00; likelihood ratio test χ^2^(1) = 0; *p* = 1.00) and was, therefore, not retained.

As a sensitivity analysis, the longitudinal mixed-effects model was refitted restricting the diabetic group to eyes with complete flare measurements at all five time points (*n* = 62 of 86 eyes; 24 eyes were excluded because of a missing measurement at week 7, week 19, or both), to examine whether restricting to complete cases altered the primary conclusions; this comparison does not by itself establish that the missing-at-random assumption holds.

### 2.9. GenAI Statement

During the preparation of this study, the author used an AI-based language assistant (Perplexity Computer; Perplexity AI, San Francisco, CA, USA) to help refine the wording of this manuscript and to draft responses to peer-review comments. The author has reviewed and edited all AI-assisted text and takes full responsibility for the scientific content, analyses, and conclusions of this publication.

## 3. Results

### 3.1. Study Population and Baseline Characteristics

A total of 172 eyes from 106 patients were included in this study, comprising 86 diabetic eyes and 86 matched control eyes. Baseline demographic and clinical characteristics are summarized in Table 1. There were no significant differences in age between the diabetic and control groups (75.0 ± 7.4 vs. 74.8 ± 5.8 years; patient-clustered GEE *p* = 0.790). Emery–Little grades were also comparable between groups (2.36 ± 0.35 vs. 2.37 ± 0.34; *p* = 0.979). The proportion of male patients was significantly higher in the diabetic group than in the control group (34/19 vs. 20/33; *p* = 0.011). Preoperative aqueous flare values were significantly higher in diabetic eyes than in control eyes (11.1 ± 6.0 vs. 7.4 ± 1.8 ph/ms; patient-clustered GEE *p* < 0.001). Diabetic eyes showed numerically higher preoperative CCT values (539 ± 38 vs. 527 ± 30 µm) and CV values (40.7 ± 7.4 vs. 38.7 ± 5.4), and slightly worse preoperative BCVA (0.149 ± 0.185 vs. 0.088 ± 0.183 logMAR); however, none of these differences remained statistically significant once patient clustering was accounted for (patient-clustered GEE: CCT *p* = 0.065, CV *p* = 0.056, and BCVA *p* = 0.057). Among diabetic eyes, 90.7% had no DR, and 9.3% had preproliferative changes, as summarized in Table 1. This skewed distribution toward absent or mild DR limits the generalizability of our findings to patients with more advanced stages of DR and should be considered when interpreting the results.

### 3.2. Intraoperative Surgical Parameters

Intraoperative surgical parameters are summarized in Table 2. There were no significant differences in operative time, phaco time, aspiration time, or irrigation fluid volume between the groups. Diabetic eyes showed numerically higher CDE than control eyes (6.94 ± 2.51 vs. 6.16 ± 1.77), but this difference did not reach statistical significance once patient clustering was accounted for (patient-clustered GEE *p* = 0.051).

### 3.3. Postoperative Aqueous Flare Measurements

**Figure 2:**
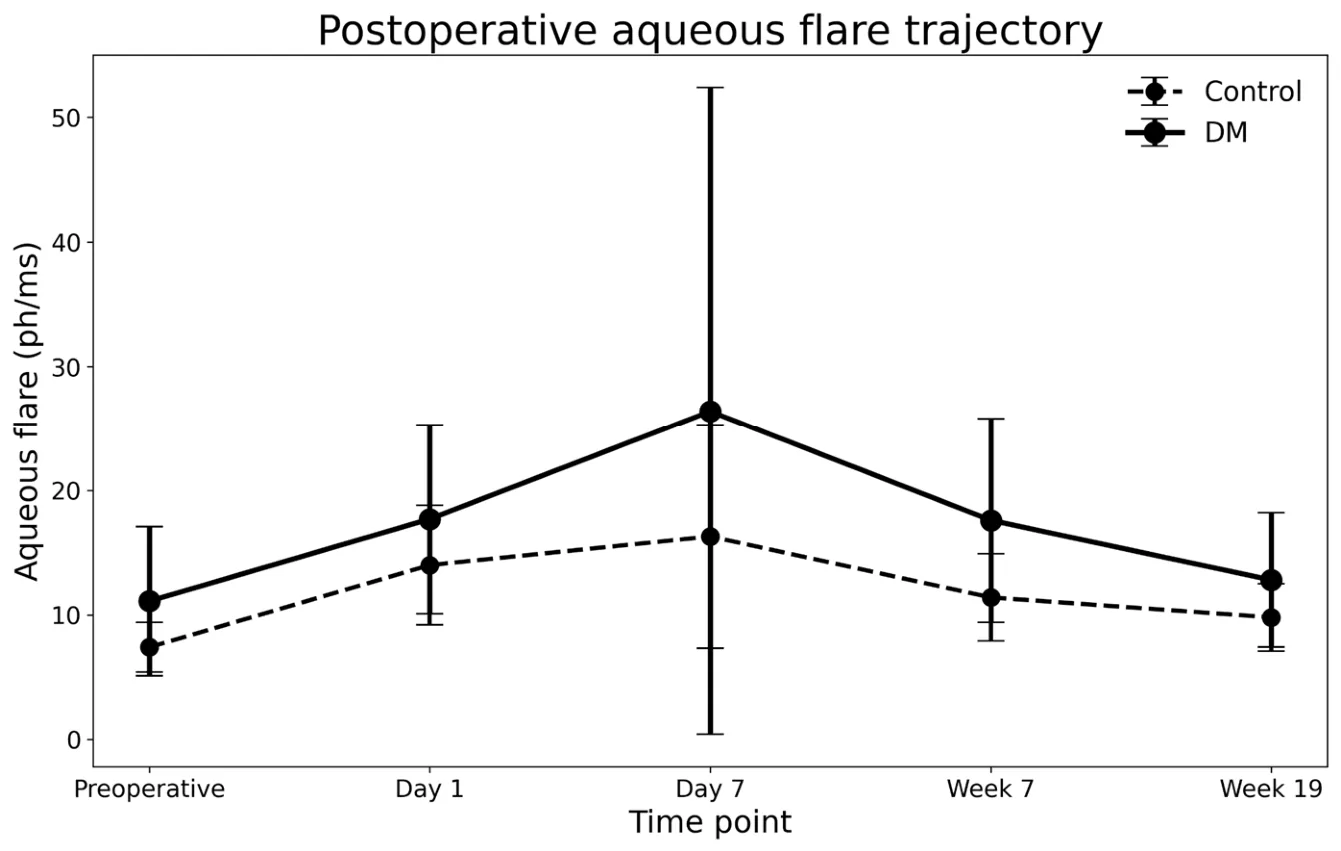
Postoperative aqueous flare trajectory in DM and control eyes. Data are presented as means ± standard deviation (SD). Sample sizes: control group, *n* = 86 eyes at all time points; DM group, *n* = 86 eyes at preoperative, day 1, and day 7 time points, *n* = 75 eyes at week 7, and *n* = 66 eyes at week 19, reflecting missed scheduled visits at these time points (Section 2.2). Between-group comparisons at each time point were statistically significant (patient-clustered GEE *p* < 0.001 at all time points except postoperative day 7 (*p* = 0.004); see Table 3). Individual data points are provided in Supplementary File S1. DM, diabetes mellitus.

Longitudinal aqueous flare measurements are summarized in Table 3 and illustrated in Figure 2. In control eyes, aqueous flare values increased from 7.4 ± 1.8 ph/ms preoperatively to 14.0 ± 4.6 ph/ms on postoperative day 1 and 16.3 ± 9.0 ph/ms on postoperative day 7, followed by gradual decreases at postoperative week 7 and week 19. In diabetic eyes, aqueous flare values increased from 11.1 ± 6.0 ph/ms preoperatively to 17.7 ± 7.6 ph/ms on postoperative day 1 and 26.4 ± 25.9 ph/ms on postoperative day 7, followed by gradual decreases at postoperative week 7 and week 19. Postoperative flare values were significantly higher in diabetic eyes than in control eyes at all postoperative time points, including postoperative day 1, postoperative week 7, and postoperative week 19 (all patient-clustered GEE *p* < 0.001), and postoperative day 7 (patient-clustered GEE *p* = 0.004). The greatest between-group difference was observed at postoperative day 7. When expressed as fold-changes from baseline, the day 7 flare increased to 2.26-fold in control eyes and 2.64-fold in diabetic eyes, with no significant between-group difference (*p* = 0.22). Among diabetic eyes, in most cases, the patient did not attend the scheduled postoperative visit at one or more time points, resulting in missing data for all assessments performed at that visit, including aqueous flare; in a small number of cases, only some assessments, including in one instance of aqueous flare alone, were not obtained despite the visit being attended (11 of 86 eyes affected at week 7 and a cumulative 20 of 86 eyes at week 19). These missed visits and omissions were not related to postoperative complications or adverse events, and no imputation was performed; the linear mixed-effects models used all available measurements at each time point.

### 3.4. Postoperative Clinical Outcomes

Postoperative clinical outcomes are summarized in Table 4. At postoperative week 7, CCT values remained significantly higher in diabetic eyes than in control eyes (544 ± 40 vs. 530 ± 30 µm; patient-clustered GEE *p* = 0.018). At postoperative week 19, diabetic eyes continued to demonstrate significantly higher IOP values (13.0 ± 2.2 vs. 12.1 ± 1.8 mmHg; patient-clustered GEE *p* = 0.011) and CCT values (540 ± 37 vs. 528 ± 31 µm; patient-clustered GEE *p* = 0.031). Postoperative CECD loss (%) differed significantly between groups at both week 7 (control 1.50 ± 2.35% vs. DM −0.35 ± 3.30%; *p* < 0.001) and week 19 (control 1.44 ± 2.25% vs. DM −0.38 ± 4.76%; *p* = 0.005), with diabetic eyes showing an apparent increase in CECD relative to preoperative values. No intraoperative complications or postoperative adverse events requiring exclusion from the analyses were observed in this cohort; the reduced number of diabetic eyes analyzed at week 7 and week 19 (Table 4) reflects missed scheduled visits, as described in Section 2.2, rather than any complication or adverse event.

### 3.5. Primary Multivariable Analysis of Postoperative Day 7 Aqueous Flare

The primary analysis of factors associated with postoperative day 7 aqueous flare was a linear mixed-effects model with log-transformed day 7 flare as the outcome; diabetes mellitus group, preoperative flare, cumulative dissipated energy, irrigation fluid volume, and operative time as fixed effects; and patient ID as a random intercept to account for correlation between bilateral eyes (Table 5). In this model, diabetic status remained significantly associated with approximately 25% higher postoperative day 7 flare values (exp(β) = 1.25; 95% CI: 1.03–1.53; *p* = 0.026). Preoperative flare (*p* < 0.001) and intraoperative irrigation fluid volume (*p* = 0.020) were also significantly associated with postoperative flare, whereas cumulative dissipated energy (*p* = 0.619) and operative time (*p* = 0.345) were not independently associated. VIFs for the predictors retained in this model ranged from 1.11 to 2.62, with the highest values observed for irrigation fluid volume (VIF = 2.62) and operative time (VIF = 2.22), reflecting a strong positive correlation between these two variables (*r* = 0.71); these values remain below conventional thresholds for problematic multicollinearity. The variance components of the model indicated substantial between-patient variability relative to residual variance, corresponding to an ICC of approximately 0.75, reflecting a strong correlation between fellow eyes.

As a sensitivity analysis, we refit the primary log-transformed model (Table 5) with sex added as a covariate and assessed whether the diabetes coefficient changed materially after adjustment. The diabetes coefficient changed minimally after adding sex (*β* = 0.227 (*p* = 0.026) in the primary model vs. β = 0.217 (*p* = 0.038) with sex included; relative change ≈ 4.5%), while sex itself was not significantly associated with day-7 flare (*β* = 0.047; *p* = 0.639). This negligible change in the diabetes coefficient, rather than the nonsignificant sex coefficient alone, indicates that the sex imbalance between groups did not materially confound the primary findings. These findings suggest that diabetic status, preoperative BAB condition, and intraoperative irrigation fluid volume were associated with elevated postoperative aqueous flare following cataract surgery [16]; as an observational association, this finding does not establish a causal effect of fluidic stress.

### 3.6. Longitudinal Linear Mixed-Effects Model

Both group (F(1, 100.5) = 30.73; *p* < 0.001) and time (F(4, 714.9) = 140.16; *p* < 0.001) were significantly associated with flare, whereas the group × time interaction was not statistically significant (F(4, 714.9) = 2.04; *p* = 0.087), indicating no strong evidence that the magnitude of the between-group difference changed over time (Table 6). To examine whether these findings were materially affected by missing follow-up data, a sensitivity analysis restricted to diabetic eyes with complete flare measurements at all five time points (*n* = 62 of 86 eyes) yielded materially similar results: both group (F(1, 88.6) = 27.34; *p* < 0.001) and time (F(4, 641.4) = 128.61; *p* < 0.001) remained significantly associated with flare, and the group × time interaction remained nonsignificant (F(4, 641.4) = 1.09; *p* = 0.363), consistent with the primary model. Between-group differences at each time point were consistent in direction and magnitude with those from the primary analysis (Table 6), indicating that these findings were not materially altered when restricting to eyes with complete follow-up.

## 4. Discussion

The present study quantitatively evaluated postoperative inflammatory responses after cataract surgery using the Eight-Chop Technique by LFP and demonstrated significantly increased postoperative aqueous flare values in diabetic eyes compared with nondiabetic eyes. The between-group difference was most prominent at postoperative day 7, and multivariable analyses indicated that diabetic status, preoperative flare, and intraoperative irrigation fluid volume were independently associated with postoperative day 7 flare, whereas cumulative dissipated energy and operative time were not. These findings suggest that diabetic eyes maintain a persistently higher absolute level of postoperative aqueous flare across the observation period, even under contemporary low-stress phacoemulsification conditions, and that BAB dysfunction continues to play an important role [2,3,4] in diabetic eyes despite advances in cataract surgery technology. These clinical observations are consistent with experimental data indicating that diabetes-related microvascular vulnerability and chronic low-grade inflammatory activation in ocular tissues render the blood–aqueous and blood–retinal barriers more susceptible to mechanical and inflammatory stress [16].

Previous studies using LFP have similarly demonstrated prolonged postoperative inflammation in diabetic eyes after phacoemulsification [2,3,4]. Liu et al. reported significantly increased postoperative flare values in diabetic eyes, particularly in eyes with DR, suggesting persistent postoperative BAB dysfunction in diabetic patients [2]. In the present study, postoperative flare values in diabetic eyes also remained elevated at day 7, consistent with previous reports. However, the absolute flare values observed in the current study were comparatively modest relative to earlier studies evaluating conventional phacoemulsification [2,5]. Previous studies reported marked day 1 flare elevation in diabetic eyes under longer procedures with higher irrigation fluid volumes, whereas surgery in the present study was shorter and used less irrigation fluid. Moreover, absolute flare values continued to increase from postoperative day 1 to day 7 in both groups, with a numerically larger absolute increase observed in diabetic eyes; however, the relative (fold-change) increase from baseline did not differ significantly between groups (Section 3.3). These findings may suggest that immediate postoperative surgical trauma was attenuated under modern low-stress surgical conditions, while a persistently elevated absolute flare level was maintained in diabetic eyes throughout the observation period.

Active-fluidics technology enables stable anterior chamber maintenance even under relatively low intraoperative IOP conditions and may contribute to reduced intraoperative mechanical stress [17,18]. Nevertheless, the present findings indicate that postoperative inflammatory responses in diabetic eyes persist despite these contemporary refinements.

An important feature of the present study is the focus on postoperative day 7 flare values. Previous studies have suggested that flare immediately after surgery mainly reflects acute surgical trauma, whereas flare at approximately 1 week more closely reflects postoperative BAB dysfunction and inflammatory persistence [2,3]. In the present study, day 7 represented the time point at which the absolute between-group difference in flare was greatest. Flare values increased further from day 1 to day 7 in both groups, and this relative increase was similar between groups (Section 3.3), consistent with a persistently elevated absolute flare level in diabetic eyes rather than a differential postoperative trajectory. Furthermore, day 7 approximately corresponds to the timing at which postoperative topical steroid regimens are often tapered in routine clinical practice. Therefore, postoperative day 7 flare evaluation may represent a useful indicator for identifying diabetic eyes with persistently elevated inflammatory activity, although its direct association with visual or clinical outcomes was not evaluated in this study.

Another important observation was that irrigation fluid volume showed a modest association with postoperative day 7 flare values, while cumulative dissipated energy and operative time were not identified as independent factors in multivariable analyses. Traditionally, surgical invasiveness during phacoemulsification has been assessed mainly by ultrasound energy usage and operative duration [19,20]. However, recent evidence indicates that anterior chamber stability and fluidics-related intraoperative conditions substantially influence intraocular tissue stress [17,18], and our findings suggest that postoperative inflammation in contemporary cataract surgery may be more closely associated with overall fluidics-related surgical parameters, including irrigation fluid volume and anterior chamber stability, than with ultrasound energy or operative time alone.

Under contemporary small-incision phacoemulsification with efficient nucleus fragmentation strategies such as the Eight-Chop Technique, ultrasound energy is typically delivered in a more localized and controlled fashion, and the total CDE is lower than in older techniques. In this setting, fluidics-related factors, including irrigation and aspiration flow, vacuum, and anterior chamber turbulence, may play a more prominent role than ultrasound energy alone in disrupting the BAB [21,22,23]. High flow rates and large cumulative irrigation fluid volumes can increase turbulence in the anterior chamber, promote rebound of lens fragments and instruments toward the corneal endothelium and iris, and mechanically disturb endothelial and iris surfaces [21,22,23]. Such turbulence and mechanical stress are thought to damage the endothelial glycocalyx and tight junctions and to facilitate the release of inflammatory mediators into the aqueous, thereby contributing to increased flare [21,22,23]. In our study, irrigation fluid volume may, therefore, serve as a surrogate marker for cumulative fluidics-related surgical exposure within the anterior chamber, potentially reflecting other unmeasured parameters such as aspiration turbulence and transient anterior chamber instability. The finding that irrigation fluid volume, rather than CDE, showed a more consistent association with day-7 flare is in line with experimental and computational fluid dynamics studies demonstrating that irrigation/aspiration settings influence flow fields, corneal deformation, and turbulence in the anterior chamber, and are linked to endothelial and anterior segment stress.

The Eight-Chop Technique is a “segmentation-first” nuclear fragmentation strategy that completes full-thickness nuclear division before phacoemulsification, thereby simplifying intraocular manipulation compared with conventional fragmentation techniques [12,24,25]. Eight-segment nuclear division optimizes fragment geometry and aspiration efficiency and may reduce mechanical stress on intraocular tissues, particularly when combined with active-fluidics systems [12,13]. The comparatively modest postoperative flare values observed in the present study may partly reflect reduced intraoperative stress associated with this fragmentation strategy [13].

Particularly under modern active-fluidics environments, fragment geometry and mobility may substantially influence anterior chamber stability [18,21,26]. Small and uniform nuclear fragments may facilitate stable occlusion and smoother aspiration, reducing surge and unnecessary intraocular turbulence [27]. The Eight-Chop Technique was specifically designed to be compatible with contemporary low-IOP fluidics environments [13], and the present findings may support the concept that optimizing fragmentation strategy and fluidics compatibility contributes to reduced postoperative inflammation. More broadly, contemporary anterior-segment techniques increasingly seek to minimize intraocular manipulation, and Eight-Chop is consistent with this trend toward refined, less disruptive intraocular maneuvers [28].

Recent studies have further suggested that aqueous flare reflects intraocular inflammatory cytokine activity in diabetic eyes and may serve as a non-invasive biomarker of intraocular immune status [10]. Therefore, the persistently elevated postoperative flare observed in diabetic eyes in the present study may reflect ongoing inflammatory activation rather than merely residual mechanical surgical trauma. Even under modern low-stress surgical conditions, diabetic eyes may remain susceptible to persistently elevated inflammatory activity because of persistent BAB dysfunction and underlying diabetic ocular microvascular abnormalities.

Because corneal endothelial cells are generally considered nonregenerative in vivo [29], these changes most likely reflect measurement variability inherent to non-contact specular microscopy, including differences in image acquisition and automated cell recognition, as reported previously [30,31,32]. Accordingly, CECD findings in this study should be interpreted primarily as supportive safety data rather than as a main outcome measure.

Several limitations should be acknowledged. First, this was a prospective single-center observational study rather than a randomized controlled trial. As with any observational study, the associations reported here—including that between irrigation fluid volume and postoperative flare—cannot establish causality; further experimental or mechanistic studies, such as anterior chamber fluid dynamics modeling or biomarker-based approaches, are warranted to clarify the causal pathways underlying these observations. In the day 7 mixed-effects model (Table 5), VIFs ranged from 1.11 to 2.62, with the highest values observed for irrigation fluid volume (VIF = 2.62) and operative time (VIF = 2.22), reflecting a strong positive correlation between these two variables (*r* = 0.71); irrigation fluid volume was also very strongly correlated with aspiration time (*r* = 0.94) and phacoemulsification time (*r* = 0.83). Although these VIFs remain below conventional thresholds for problematic multicollinearity, the observed association between irrigation fluid volume and postoperative flare should be interpreted as an observational association rather than evidence of a causal effect of fluidic stress, given its correlation with other measures of operative duration and complexity. In addition, the control group was matched to the diabetic group by age and Emery–Little grade but not by sex, resulting in a higher proportion of male patients in the diabetic cohort. Although sex-related differences in inflammatory responses could theoretically confound aqueous flare, the diabetes coefficient changed minimally (relative change ≈ 4.5%) after adding sex to the primary model (Section 3.5), suggesting that this imbalance had a limited impact on the main conclusions. Second, all surgeries were performed by a single surgeon, which may limit generalizability to other surgical settings. Third, detailed stratification of retinopathy severity was not performed. In this cohort, most diabetic eyes had no DR (78/86; 90.7%) and only 8/86 (9.3%) had preproliferative DR, with no simple or proliferative DR, distributed across only six patients; any inferential analysis of DR status in this cohort would, therefore, be severely underpowered and highly sensitive to individual observations. For this reason, we did not include DR status as a covariate in the primary multivariable model and do not report inferential DR-stratified estimates in this manuscript. The generalizability of our findings to patients with more advanced stages of DR remains limited, and further studies in cohorts with a broader spectrum of DR severity are required to clarify how diabetic retinal disease severity influences postoperative inflammatory responses. Nevertheless, the present study also has important strengths, including use of a relatively uniform surgical environment with a single fragmentation strategy and fluidics platform, objective quantitative evaluation using LFP, and longitudinal statistical analysis using mixed-effects models to appropriately account for inter-eye dependency [33,34,35].

## 5. Conclusions

Diabetic eyes demonstrated a persistently higher absolute level of postoperative aqueous flare compared with control eyes across all postoperative time points, even under contemporary low-stress cataract surgery conditions using the Eight-Chop Technique, without evidence that the magnitude of this difference changed disproportionately over time (group × time interaction; *p* = 0.087). Postoperative day 7 aqueous flare may represent a useful indicator of persistent BAB dysfunction in diabetic eyes, although its association with clinical outcomes was not directly evaluated. In addition, the association between irrigation fluid volume and postoperative flare suggests that fluidics-related surgical parameters may be associated with postoperative inflammation during modern phacoemulsification surgery, though this observational association does not establish causality.

## Figures and Tables

**Table 1:** Baseline characteristics of diabetic and control eyes.

Variable	Control (*n* = 86 Eyes)	DM (*n* = 86 Eyes)	*p*-Value
Number of eyes	86	86	NA
Number of patients	53	53	NA
Age, years	74.8 ± 5.8	75.0 ± 7.4	0.790
Sex, male/female (patients)	20/33	34/19	0.011
Emery–Little grade	2.37 ± 0.34	2.36 ± 0.35	0.979
Preoperative BCVA (logMAR)	0.088 ± 0.183	0.149 ± 0.185	0.057
Preoperative IOP (mmHg)	13.8 ± 1.7	14.1 ± 1.8	0.246
Preoperative CECD (cells/mm^2^)	2659 ± 187	2645 ± 283	0.708
Preoperative CCT (µm)	527 ± 30	539 ± 38	0.065
Preoperative CV	38.7 ± 5.4	40.7 ± 7.4	0.056
Preoperative PHC (%)	46.0 ± 7.1	44.0 ± 7.8	0.083
Preoperative flare (ph/ms)	7.4 ± 1.8	11.1 ± 6.0	<0.001
HbA1c (%)	5.6 ± 0.3	6.8 ± 0.9	<0.001
DR stage, *n* (%)			
No DR	NA	78 (90.7)	NA
Simple DR	NA	0 (0.0)	NA
Preproliferative DR	NA	8 (9.3)	NA

Values are presented as means ± SD or number (%), unless otherwise indicated. *p*-values for continuous variables were derived from patient-clustered generalized estimating equations (GEEs; exchangeable correlation structure, clustering by patient) to account for correlation between bilateral eyes of the same patient. The Emery–Little grade was compared using the Mann–Whitney U test, and sex was compared using Fisher’s exact test, both at the patient level. NA indicates that a statistical comparison was not performed (e.g., for descriptive counts and DR stage subcategories). DR stage is shown descriptively for diabetic eyes only. “Any DR” was defined as the presence of simple or preproliferative DR; in this cohort, only preproliferative DR contributed to the “any DR = 1” category. BCVA, best-corrected visual acuity; IOP, intraocular pressure; CECD, corneal endothelial cell density; CCT, central corneal thickness; CV, coefficient of variation; PHC, percentage of hexagonal cells; DR, diabetic retinopathy; DM, diabetes mellitus; logMAR, logarithm of the minimum angle of resolution.

**Table 2:** Intraoperative surgical parameters.

Variable	Control (*n* = 86 Eyes)	DM (*n* = 86 Eyes)	*p*-Value
Operative time (min)	5.2 ± 1.0	5.5 ± 1.3	0.196
Phaco time (s)	17.3 ± 6.4	18.7 ± 7.7	0.270
Aspiration time (s) (DM *n* = 85)	79.2 ± 20.7	82.2 ± 26.3	0.471
Cumulative dissipated energy	6.16 ± 1.77	6.94 ± 2.51	0.051
Irrigation fluid volume (mL)	29.3 ± 8.9	30.5 ± 11.7	0.502

Values are presented as means ± SD. *p*-values were derived from patient-clustered generalized estimating equations (GEEs; exchangeable correlation structure, clustering by patient) to account for correlation between bilateral eyes of the same patient. Cumulative dissipated energy is a unitless index calculated as average phaco power (%) × ultrasound time (seconds); it does not represent an absolute energy value (e.g., joules).

**Table 3:** Longitudinal aqueous flare measurements.

Variable	Control	DM	*p*-Value
Preoperative flare (ph/ms) (Control *n* = 86; DM *n* = 86)	7.4 ± 1.8; median: 7.2 [6.1–8.5]	11.1 ± 6.0; median: 9.6 [7.1–12.1]	<0.001
Postoperative day 1 flare (ph/ms) (Control *n* = 86; DM *n* = 86)	14.0 ± 4.6; median: 13.1 [10.7–16.4]	17.7 ± 7.6; median: 15.7 [12.5–20.8]	<0.001
Postoperative day 7 flare (ph/ms) (Control *n* = 86; DM *n* = 86)	16.3 ± 9.0; median: 14.0 [11.5–17.9]	26.4 ± 25.9; median: 18.0 [13.4–25.3]	0.004
Postoperative week 7 flare (ph/ms) (Control *n* = 86; DM *n* = 75)	11.4 ± 3.6; median: 11.0 [8.8–13.1]	17.5 ± 8.3; median: 15.1 [12.0–20.1]	<0.001
Postoperative week 19 flare (ph/ms) (Control *n* = 86; DM *n* = 66)	9.7 ± 2.8; median: 9.1 [7.7–11.0]	12.8 ± 5.3; median: 12.4 [9.1–14.1]	<0.001

Values are presented as means ± SD; medians [IQR] are also shown, given the markedly right-skewed distribution of flare values. *p*-values were derived from patient-clustered generalized estimating equations (GEEs; exchangeable correlation structure, clustering by patient) to account for correlation between bilateral eyes of the same patient.

**Table 4:** Postoperative clinical outcomes.

Variable	Control	DM	*p*-Value
Postoperative week 7 BCVA (logMAR) (Control *n* = 86; DM *n* = 76)	−0.062 ± 0.037	−0.048 ± 0.066	0.168
Postoperative week 7 IOP (mmHg) (Control *n* = 86; DM *n* = 76)	12.2 ± 2.1	12.7 ± 1.7	0.100
Postoperative week 7 CCT (µm) (Control *n* = 86; DM *n* = 75)	530 ± 30	544 ± 40	0.018
Postoperative week 7 CECD loss (%) (Control *n* = 86; DM *n* = 75)	1.50 ± 2.35	−0.35 ± 3.30	<0.001
Week 19 (Control *n* = 86; DM *n* = 66)			
Postoperative week 19 BCVA (logMAR)	−0.065 ± 0.030	−0.055 ± 0.058	0.291
Postoperative week 19 IOP (mmHg)	12.1 ± 1.8	13.0 ± 2.2	0.011
Postoperative week 19 CCT (µm)	528 ± 31	540 ± 37	0.031
Postoperative week 19 CECD loss (%)	1.44 ± 2.25	−0.38 ± 4.76	0.005

Values are presented as means ± SD. *p*-values were derived from patient-clustered generalized estimating equations (GEEs; exchangeable correlation structure, clustering by patient) to account for correlation between bilateral eyes of the same patient. BCVA, best-corrected visual acuity; IOP, intraocular pressure; CCT, central corneal thickness; CECD, corneal endothelial cell density; DM, diabetes mellitus; logMAR, logarithm of the minimum angle of resolution; SD, standard deviation.

**Table 5:** Linear mixed-effects model for postoperative day 7 aqueous flare.

Variable	Estimate	SE	df	*t*-Value	*p*-Value	95% CI Low	95% CI High
Intercept	2.394	0.173	147.9	13.838	<0.001	2.055	2.734
Diabetes mellitus group vs. control	0.227	0.100	110.7	2.259	0.026	0.030	0.424
Preoperative flare (ph/ms)	0.029	0.009	165.8	3.360	<0.001	0.012	0.045
Cumulative dissipated energy	−0.008	0.017	112.8	−0.499	0.619	−0.041	0.024
Irrigation fluid volume (mL)	0.011	0.005	106.4	2.364	0.020	0.002	0.020
Operative time (min)	−0.035	0.037	110.3	−0.949	0.345	−0.109	0.038

The dependent variable in this mixed-effects model was log-transformed postoperative day-7 aqueous flare. Coefficients are reported on the log scale; exp(coefficient) represents the multiplicative change in flare associated with each predictor. The model included patient ID as a random intercept to account for inclusion of bilateral cases. Variance components (patient-level variance of 0.19 and residual variance of 0.06) were used to estimate the intraclass correlation coefficient (ICC ≈ 0.75), which indicated a strong correlation between fellow eyes. CI, confidence interval; SE, standard error.

**Table 6:** Estimated group differences in aqueous flare (diabetes mellitus vs. control) across postoperative time points.

Time Point	Estimate (Log Scale) ^a^	SE	95% CI Low	95% CI High	*p*-Value	Fold-Change (DM/Control)
Preoperative	0.338	0.069	0.202	0.474	<0.001	1.40
Day 1	0.207	0.069	0.070	0.343	0.003	1.23
Day 7	0.335	0.069	0.199	0.471	<0.001	1.40
Week 7	0.380	0.071	0.241	0.519	<0.001	1.46
Week 19	0.268	0.072	0.127	0.410	<0.001	1.31

^a^ Estimates represent the diabetes mellitus group minus control group difference in natural-log-transformed flare; positive values indicate higher flare in the diabetes mellitus group. Fold-change = exp(estimate). Estimates derive from a linear mixed-effects model (log flare ~ Group × Time + (1 | PatientID)) fitted across all five time points, with contrasts computed using estimated marginal means (Kenward–Roger degrees of freedom). DM, diabetes mellitus; CI, confidence interval; SE, standard error.

## Data Availability

The original contributions presented in this study are included in this article/Supplementary Material. Further inquiries can be directed to the corresponding author.

## Supplementary Materials

The following supporting information can be downloaded at https://www.mdpi.com/article/10.3390/jcm15176547/s1. Supplementary File S1: Anonymized original dataset for the diabetes flare study (Diabetes Flare study original data-dmr); Supplementary File S2: Representative R code and model formulas for the multivariable regression and linear mixed-effects models.

## Abbreviations

The following abbreviations are used in this manuscript:

BAB	Blood–aqueous barrier
BCVA	Best-corrected visual acuity
CCT	Central corneal thickness
CDE	Cumulative dissipated energy
CECD	Corneal endothelial cell density
CI	Confidence interval
CV	Coefficient of variation
DM	Diabetes mellitus
DR	Diabetic retinopathy
ICC	Intraclass correlation coefficient
IOP	Intraocular pressure
LFP	Laser flare photometry
logMAR	Logarithm of the minimum angle of resolution
ph/ms	Photon counts per millisecond
PHC	Percentage of hexagonal cells
VIF	Variance inflation factor
