# Supplementary material for: Postoperative Aqueous Flare in Diabetic Eyes and Fluidics-Related Surgical Parameters After Cataract Surgery Using the Eight-Chop Technique
Source: J Clin Med. 2026 Aug 25;15(17):6547. doi: 10.3390/jcm15176547 (PMC13565849; doi:10.3390/jcm15176547)
Supplement: Supplementary file 1 [file jcm-15-06547-s001.zip › Supplementary File S2. Statistical model specifications and R code (1).pdf]

Supplementary File 2: Statistical model specifications and R code

1. Dataset structure and cohort definition

The accompanying dataset ("Diabetes_Flare_max") contains 186 eyes. To allow independent reproduction of all patient-clustered and mixed-effects analyses reported in the manuscript, the dataset includes the following identifier and inclusion variables:

- PatientID – an anonymous patient-level cluster identifier, repeated for both eyes of the same patient. This is the clustering variable used as the random-intercept grouping factor in all mixed-effects models and as the cluster identifier in all generalized estimating equations (GEE) models.
- EyeID – an eye-level identifier (1 or 2) distinguishing the two eyes of the same patient; PatientID combined with EyeID uniquely identifies each of the 186 eyes.
- Included_172 – a logical analysis-inclusion flag (TRUE/FALSE). This equals TRUE for the 172 eyes (86 control, 86 diabetic) analyzed in the manuscript, and FALSE for the 14 eyes excluded because of missing postoperative follow-up (Group = "Missing_FollowUp") or because they were not selected during age/Emery–Little Grade matching (Group = "Not_Selected_Matching").
- Group – a four-level variable ("control", "DM", "Missing_FollowUp", "Not_Selected_Matching") describing every eye in the raw 186-eye dataset, retained for transparency.

2. Cohort filtering and derived-variable creation

All analyses in Sections 3–6 below were performed on the filtered 172-eye cohort, created from the raw 186-eye dataset as follows. This code block is a required first step and must be run before any model in this file.

R code:

```
# Filter to the 172 eyes (106 patients) analyzed in the manuscript
df_172 <- subset(Diabetes_Flare_max, Included_172 == TRUE)
df_172$Group <- factor(df_172$Group, levels = c("control", "DM"))
df_172 <- df_172[order(df_172$PatientID), ]

# Derived outcome variable used in the GEE, primary, and longitudinal models
df_172$log_D7_Flare <- log(df_172$D7_Flare_photons_ms)

# Confirms 172 eyes from 106 patients
nrow(df_172)
length(unique(df_172$PatientID))
```

3. Patient-clustered GEE models for descriptive comparisons (Tables 1–4)

To account for correlation between bilateral eyes of the same patient, p-values for continuous variables in the descriptive comparison tables (Tables 1–4) were derived from generalized estimating equations (GEE) with an exchangeable working correlation structure, clustering by PatientID, rather than from unadjusted Welch's t-tests.

R code:

```
library(geepack)

get_clustered_p_continuous <- function(varname, data) {
  f <- as.formula(paste0(varname, " ~ Group"))
  m <- geeglm(f, id = PatientID, data = data, family = gaussian, corstr =
```

```
"exchangeable")
  s <- summary(m)$coefficients
  data.frame(variable = varname, estimate = s["GroupDM", "Estimate"],
             p_value = s["GroupDM", "Pr(>|W|)"])
}

# Example: Table 1, preoperative central corneal thickness
get_clustered_p_continuous("Pre_CCT_um", df_172)

# Applied identically to all continuous variables in Tables 1–4
# (e.g., Pre_BCVA_logMAR, Pre_CV, Pre_IOP_mmHg, Pre_CECD_cells_mm2,
#  OperativeTime_min, PhacoTime_sec, AspirationTime_sec, CDE, FluidVolume_mL,
#  W7_BCVA_logMAR, W7_IOP_mmHg, W7_CCT_um, CECD_loss_W7,
#  W19_BCVA_logMAR, W19_IOP_mmHg, W19_CCT_um, CECD_loss_W19)
```

Sex is a patient-level, not eye-level, covariate (identical for both eyes of the same patient) and was therefore compared using Fisher's exact test at the patient level; the Emery–Little Grade was compared using the Mann–Whitney U test. Neither requires clustering correction.

4. Linear mixed-effects model for postoperative day-7 aqueous flare (Table 5)

The linear mixed-effects model summarized in Table 5 was specified as:

Model formula:

```
log_D7_Flare = β0 + β1*Group + β2*Pre_Flare + β3*CDE +
               β4*FluidVolume + β5*OperativeTime + u_patient + ε
```

where u_patient ~ N(0, σ_patient²) is a random intercept for patient ID.

R code:

```
library(lme4)
library(lmerTest)

lmm_model <- lmer(
  log_D7_Flare ~ Group + Pre_Flare_photons_ms + CDE +
    FluidVolume_mL + OperativeTime_min + (1 | PatientID),
  data = df_172
)
summary(lmm_model)
confint(lmm_model, method = "Wald")
```

As a sensitivity analysis, this model was refitted with sex added as an additional covariate to assess whether the diabetes coefficient changed materially after adjustment (Section 3.5):

```
model_sex <- lmer(
  log_D7_Flare ~ Group + Sex_bin + Pre_Flare_photons_ms + CDE +
    FluidVolume_mL + OperativeTime_min + (1 | PatientID),
  data = df_172
)
summary(model_sex)

pct_change <- (fixef(model_sex)["GroupDM"] - fixef(lmm_model)["GroupDM"]) /
  fixef(lmm_model)["GroupDM"] * 100
pct_change
```

5. Longitudinal linear mixed-effects model for aqueous flare across all postoperative time points (Table 6)

To evaluate whether the between-group difference in aqueous flare changed over time, a linear mixed-effects model was fitted to natural-log-transformed flare values at all five time points (preoperative, day 1, day 7, week 7, and week 19), with fixed effects for group, time, and their interaction, and a random intercept for patient ID to account for correlation between bilateral cases.

Model formula:

```
log_Flare = β0 + β1*Group + β2*Time + β3*(Group×Time) + u_patient + ε
```

R code:

```
library(dplyr)
library(tidyr)
library(lme4)
library(lmerTest)
library(emmeans)

# Reshape from wide to long format (five flare time points into one column)
df_long <- df_172 %>%
  select(PatientID, EyeID, Group,
         Pre_Flare_photons_ms, D1_Flare_photons_ms,
         D7_Flare_photons_ms, W7_Flare_photons_ms, W19_Flare_photons_ms) %>%
  pivot_longer(
    cols = ends_with("_Flare_photons_ms"),
    names_to = "Time",
    values_to = "Flare"
  ) %>%
  mutate(
    Time = recode(Time,
                  "Pre_Flare_photons_ms" = "Pre",
                  "D1_Flare_photons_ms"  = "D1",
                  "D7_Flare_photons_ms"  = "D7",
                  "W7_Flare_photons_ms"  = "W7",
                  "W19_Flare_photons_ms" = "W19"),
    Time = factor(Time, levels = c("Pre", "D1", "D7", "W7", "W19"))
  )

df_long_model <- df_long %>% filter(!is.na(Flare))
df_long_model$log_Flare <- log(df_long_model$Flare)

# Group x Time interaction model, random intercept for patient ID
model_longitudinal <- lmer(
  log_Flare ~ Group * Time + (1 | PatientID),
  data = df_long_model,
  REML = TRUE
)

summary(model_longitudinal)
anova(model_longitudinal)
confint(model_longitudinal, method = "Wald")

# Between-group difference at each time point
emm <- emmeans(model_longitudinal, ~ Group | Time)
pairs(emm, adjust = "none")
confint(pairs(emm, adjust = "none"))
```

Note: A three-level structure additionally nesting eye within patient, (1 | PatientID/EyeID), was also tested but did not improve model fit (ΔAIC = 2.00; likelihood ratio test $\chi^2(1) = 0$, p = 1.00) and was therefore not retained; the two-level random-intercept model above was used as the final model.

6. Sensitivity analysis: complete-case subset restricted to eyes with flare data at all five time points

To assess the robustness of the longitudinal model (Table 6) to missing postoperative data, the model was refitted in the subset of eyes with complete flare measurements at all five time points (Control: n = 86 eyes; DM: n = 62 eyes).

R code:

```
# Retain only eyes with data at all five time points
complete_eyes <- df_long_model %>%
  group_by(PatientID, EyeID) %>%
  filter(n() == 5) %>%
  ungroup()

complete_eyes %>% distinct(PatientID, EyeID, Group) %>% count(Group)

# Sensitivity model (same structure as the primary longitudinal model)
model_sensitivity <- lmer(log_Flare ~ Group * Time + (1 | PatientID),
                           data = complete_eyes, REML = TRUE)

anova(model_sensitivity, ddf = "Kenward-Roger")

emm_sensitivity <- emmeans(model_sensitivity, ~ Group | Time, lmer.df = "kenward-roger")
pairs(emm_sensitivity)
```

This sensitivity analysis yielded results materially similar to the primary model: the group main effect ($F(1, 88.6) = 27.34$, $p < 0.001$) and time main effect ($F(4, 641.4) = 128.61$, $p < 0.001$) remained significant, and the group × time interaction remained nonsignificant ($F(4, 641.4) = 1.09$, $p = 0.363$), consistent with the primary model ($F(4, 714.9) = 2.04$, $p = 0.087$).

Note: An earlier version of this supplementary file included an ordinary least-squares regression section (a simple lm() model for log-transformed day-7 flare). That model was not used to generate any result reported in the manuscript and has been removed.
